# Clinical Outcomes of the “Endovascular Aneurysm Repair-First” Protocol for Ruptured Abdominal Aortic Aneurysms: A 10-Year Single-Center Retrospective Study

**DOI:** 10.3400/avd.oa.26-00049

**Published:** 2026-09-11

**Authors:** Yutaka Kobayashi, Atsushi Kawakami

**Affiliations:** Department of Cardiovascular Surgery, Uji Tokushukai Medical Center, Uji, Kyoto, Japan

**Keywords:** endovascular aneurysm repair, abdominal aortic aneurysm, retrospective study, endovascular procedures, ruptured abdominal aortic aneurysms

## Abstract

**Objectives:**

The treatment of ruptured abdominal aortic aneurysms (rAAAs) remains associated with high morbidity and mortality. While endovascular aortic repair (EVAR) has shown promising results in elective settings, the evidence supporting its use as the primary treatment for patients with rAAA remains controversial. We aimed to evaluate both the clinical outcomes and the inherent advantages and disadvantages of our “EVAR-first” protocol for patients with rAAA over a 10-year period.

**Methods:**

A retrospective review of data extracted from medical records identified 93 consecutive patients with rAAA who underwent EVAR from January 2014 to January 2024. All patients were treated using the “EVAR-first” protocol. Initially, percutaneous puncture of the bilateral common femoral arteries and the left brachial artery was performed. General anesthesia was then induced with the endovascular balloon control and EVAR was performed.

**Results:**

EVAR was successfully performed in all patients with rAAA. The mean follow-up period was 1386.5 ± 1171.1 days. After the 30-day period, no new complications were observed. Endovascular re-interventions were performed in 9 patients (9.7%). The overall survival rates at 30 days, 1 year, and 3 years for the entire cohort were 87.1%, 70.9%, and 60.5%, respectively.

**Conclusions:**

This single-center experience suggests favorable outcomes for adopting the “EVAR-first” policy for all rAAA patients.

## Introduction

Treatment of ruptured abdominal aortic aneurysms (rAAAs) remains associated with high morbidity and mortality, despite improvements in 30-day mortality, which is now reported to be approximately 30%–35%.^1–2)^ While endovascular aortic repair (EVAR) has shown promising results in elective settings, the evidence supporting its use as the primary treatment for patients with rAAA remains controversial.^3)^ On April 21, 1994, Marin et al.^4)^ reported the first endovascular repair of rAAA (rEVAR). Since then, endovascular approaches to rAAA have become more widespread. At many institutions, rEVAR has yielded positive outcomes by reducing perioperative mortality.^5–8)^ Several recent studies have demonstrated that rEVAR is associated with reduced 30-day mortality, although its impact on long-term outcomes, such as overall mortality and morbidity, is still unclear.^3,9–10)^ Several studies have shown that standard endovascular protocols have reduced in-hospital mortality rates for rAAA from approximately 50% to approximately 16%–36%.^2,11,12)^ Based on the results of these studies, recent guidelines (including European, AHA/ACC, and Japanese guidelines) recommend EVAR as the first-line treatment option for patients with rAAA and suitable anatomy.^13–15)^ This study aimed to evaluate both the clinical outcomes and the inherent advantages and disadvantages of the “EVAR-first” protocol for all patients who presented with rAAA over a 10-year period.

## Materials and Methods

### Ethics declaration

The research protocol for this single-center retrospective cohort study was reviewed and approved by the Institutional Review Board of our institution (approval number: 2018-19). The requirement for informed consent from the patients was waived. The study was performed in accordance with the Declaration of Helsinki.

### Study design and participants

A retrospective review of data extracted from medical records identified 93 consecutive patients with rAAA who underwent endovascular repair from January 2014 to January 2024 (120 months). Patient characteristics, comorbidities, intraoperative variables, and clinical outcomes were recorded. During this study period, all patients with rAAA who were transported to our emergency department were directly transferred to the operating room and managed under the “EVAR-first” protocol, regardless of their hemodynamic severity or anatomical difficulties. No patients were left untreated (managed conservatively or palliated), and there were no conversions to open surgical repair. Patients with thoracic and thoracoabdominal aortic aneurysms, as well as those with isolated common iliac artery aneurysms, were excluded. Rupture was defined as the obvious presence of blood outside the aortic wall on preoperative contrast-enhanced computed tomography (CT). The primary endpoint was rAAA-related mortality immediately after surgery and within 1 month. The secondary endpoints included AAA-related postoperative complications, such as acute limb ischemia, myocardial infarction, length of stay, abdominal compartment syndrome, renal and respiratory failure, and rAAA-related re-interventions. Preoperative shock (severe hypotension) was defined as an initial systolic blood pressure of <80 mmHg upon arrival. Abdominal compartment syndrome (ACS) was clinically diagnosed based on ventilatory distress, organ hypoperfusion, and an intra-abdominal pressure (measured via bladder pressure) exceeding 20 mmHg. Anatomical unsuitability for standard EVAR instructions for use (IFU) was defined as the presence of 1 or more of the following adverse features on preoperative contrast-enhanced CT: a short proximal aortic neck (<15 mm), severe neck angulation (>60°), or hostile iliac landing zones requiring external iliac fixation.

### Patients and follow-up

#### rEVAR-first protocol

Briefly, once the vascular surgeon accepts the patient, the patient is transported directly to the hybrid operating room. Under local anesthesia, percutaneous puncture of both common femoral arteries and the left brachial artery is performed, with ultrasound guidance if needed. Endovascular balloon control (EBC) using a 30-mm Nipro Occlusion Catheter (Nipro, Osaka, Japan) is placed under fluoroscopic guidance at the T12 or L1 level (with T12 preferred as the optimal position) through the left brachial artery. While the catheter procedure is being performed, another physician reviews the CT scan to determine the model and size of the device to be used. General anesthesia is then induced in a controlled manner. Angiography is performed through the catheter inserted from the femoral artery to confirm the indication for endovascular repair and to position and deploy the endovascular graft just below the renal artery. The main body is deployed with the EBC in place, and the EVAR is then completed as usual. If the proximal neck length is less than 8 mm, occlusion of the renal artery and proximal extension should be considered. Postoperatively, the patients are admitted to the intensive care unit, where bladder pressure is measured as needed, and muscle relaxants are administered for patient management. If the bladder pressure exceeds 20 mmHg, a retroperitoneal incision should be considered immediately. After confirming the absence of leaks and abnormal blood flow on CT, the ventilator is weaned off once intra-abdominal pressure has decreased.

### Perioperative outcomes and complications

Complications of interest included conversion of EVAR to open repair, reoperation for a type 1a leak, tracheostomy, wound dehiscence, mesenteric ischemia, pneumonia, and renal failure requiring hemodialysis. We also examined discharge disposition, whether to home or an institutional facility, and length of stay among patients who survived until hospital discharge.

### Long-term complications

Subsequent AAA-related hospitalizations, along with the associated diagnosis and procedure codes, were identified. These included open surgical re-interventions (open repair of the aneurysm, conversion from endovascular to open repair, repair of a graft–enteric fistula or graft infection, axillo-bifemoral bypass), endovascular re-interventions (repeat endovascular repair, stent graft extension, embolization, aortic or iliac angioplasty, graft thrombectomy), and laparotomy-related complications (nonsurgical bowel obstruction, surgical bowel obstruction requiring lysis of adhesions or bowel resection, and repair of incisional hernias).

### Statistical analysis

De-identified data were analyzed through a database using SPSS v30 (IBM, Chicago, IL, USA). Descriptive statistics, including the mean and standard deviation, were applied as appropriate. A t-test was used where applicable. The Kaplan–Meier method was employed to estimate survival and patency rates. To evaluate the impact of clinical and anatomical heterogeneity on long-term survival, stratified subgroup analyses (stable vs. unstable, and anatomically suitable vs. unsuitable) were performed. To identify independent predictors of long-term mortality while controlling for potential confounding factors, variables including age, preoperative shock, anatomical unsuitability, and preoperative hemoglobin level were incorporated into a multivariate Cox proportional hazards model based on their clinical relevance and previously established prognostic significance in rAAA. Furthermore, a multivariate Cox proportional hazards regression model was constructed, incorporating age, preoperative shock, anatomical unsuitability, and preoperative hemoglobin levels to identify independent predictors of long-term mortality. A p-value of <0.05 was considered statistically significant.

## Results

### Patient characteristics

A total of 93 patients with rAAA were treated during the study period, and EVAR was performed in all cases. None of the patients required conversion to open surgical repair (OSR). The mean age was 77.3 ± 9.5 (range, 51–93) years, and the majority of patients treated were men (76.3%). Relevant patient characteristics and comorbidities are summarized in **Table 1**. Overall, 11 patients who underwent rEVAR had previously received EVAR (all of whom were confirmed to have ruptured due to late Type Ia endoleak), and 1 patient had undergone OSR for an AAA. Most patients (66.7%) were diagnosed with rAAA and transferred to our hospital from a regional hospital. Perioperative characteristics of the patients are listed in **Table 2**. The average AAA size at surgery was 77.7 (range, 39–132) mm, with an average size of 79.2 (range, 40–132) mm in men and 72.6 (range, 39–100) mm in women. Of these aneurysms, 11.8% (range, 39–53) mm were less than 55 mm, with 8.7% (range, 40–50) mm in men and 3.2% (range, 39–53) mm in women. Among the 61 patients not suitable for EVAR, 21 patients (22.6%) had a short neck (<15 mm), 33 (35.5%) had an angulated neck (>60°), and 26 (28%) had external iliac landing sites. No patient underwent OSR, and an intraoperative aortic occlusion balloon was required in 69 patients (74.2%). The mean operative time was 143.1 ± 71.2 (range, 62–565) minutes. More than half of the patients presented with severe hypotension (63.4%). Nineteen (20.4%) patients were categorized into Fitzgerald’s class I, 18 (19.4%) patients into Fitzgerald’s class II, 47 (50.5%) patients into Fitzgerald’s class III, and 9 (9.7%) patients into Fitzgerald’s class IV. Preoperative hemoglobin was less than 6 g/dL in 17 (18.3%) patients.

**Table 1:** Patient demographics and comorbidities

Variable mean ± SD, or No. (%)	
EVAR	93
Open repair	0
Converted to open surgical repair	0
Age, years	77.3 ± 9.5
Male/Female	71/22
Past medical history	
Hypertension	37 (39.8)
Stroke	15 (16.1)
Post-PCI	15 (16.1)
Dyslipidemia	17 (18.3)
Diabetes	8 (8.6)
COPD	6 (6.5)
Previous history of EVAR	11 (11.8)
Previous history of open surgical repair	1 (1.1)
Transferred from a regional hospital	62 (66.7)

COPD, chronic obstructive pulmonary disease; EVAR, endovascular aortic repair; PCI, percutaneous coronary intervention, SD, standard deviation

**Table 2:** Perioperative characteristics of the patients

Variable mean ± SD, or No. (%)	
Mean AAA size, mm	77.7 ± 19.1
For men	79.2 ± 19.5
For women	72.6 ± 16.8
Less than 55 mm	11 (11.8)
In men	8 (8.7)
In women	3 (3.2)
Not suitable for EVAR	61 (65.6)
Short neck (<15 mm)	21 (22.6)
Angulation neck (>60°)	33 (35.5)
External iliac landing	26 (28)
Fitzgerald classification	
I	19 (20.4)
II	18 (19.4)
III	47 (50.5)
IV	9 (9.7)
Preoperative hemoglobin, g/dL	
<6	17 (18.3)
6–10	33 (35.5)
10<	43 (46.2)
Severe hypotension	59 (63.4)
Intraoperative aortic occlusion balloon	69 (74.2)
Device	
Endurant	48 (51.6)
Excluder	30 (32.3)
AFX	3 (3.2)
TX2	1 (1.1)
Extension only	11 (11.8)

Categories of anatomical unsuitability are not mutually exclusive; individual patients may present with multiple adverse anatomical features.

AAA, abdominal aortic aneurysm; EVAR, endovascular aortic repair; SD, standard deviation

### Early outcomes (within 30 days)

Myocardial infarction did not occur in any patient. Acute kidney dysfunction requiring dialysis was noted in 11 patients (11.8%). Prolonged intubation (>2 weeks) and ventilation occurred in 10 patients (10.8%). Other major adverse events were relatively infrequent. Leg ischemia requiring intervention did not occur, and mesenteric and colonic ischemia were reported in 2 patients (2.2%) in case of multiple organ failure. Four patients (4.3%) experienced renal artery occlusion, and each case was found to be a pararenal aneurysm. Two of the patients required use of the chimney technique for the superior mesenteric artery. These details are summarized in **Table 3**. Overall, 12 patients died postoperatively within 30 days of presentation, all of whom died within 48 hours of surgery. ACS was observed in 7 patients (7.5%), all of whom developed multiple organ failure and died within 24 hours of EVAR, representing a subset of these early mortalities. ACS was clinically identified by ventilatory distress, evidence of organ hypoperfusion in the absence of bleeding, and increased intra-abdominal pressure. Management involved intraoperative evaluation to determine the need for a retroperitoneal incision, with laparotomy performed if necessary. In most cases, patients were managed conservatively unless there was tension or suspected ongoing bleeding.

**Table 3:** Early outcomes of the “EVAR-first” protocol for ruptured abdominal aortic aneurysms

Variable mean ± SD, or No. (%)	
Myocardial infarction	0
Acute kidney injury, requiring dialysis	11 (11.8)
Prolonged intubation (>2 week)	10 (10.8)
Leg ischemia, requiring intervention	0
Mesenteric and colonic ischemia occurred	2 (2.2)
ACS	7 (7.5)
Ureteral stenosis	1 (1.1)
Reoperation for bleeding	0
SMA syndrome	1 (1.1)
Stroke	1 (1.1)
Renal artery occlusion	4 (4.3)
Discharge disposition	
Home	48 (51.6)
Institutional facility	28 (30.1)
Length of stay among patients, days	46.5 ± 56.8
30 days mortality	12 (12.9)
<48 hours	12 (12.9)

ACS, abdominal compartment syndrome; EVAR, endovascular aortic repair; SD, standard deviation; SMA, superior mesenteric artery

### Late outcomes

**Fig. 1:**
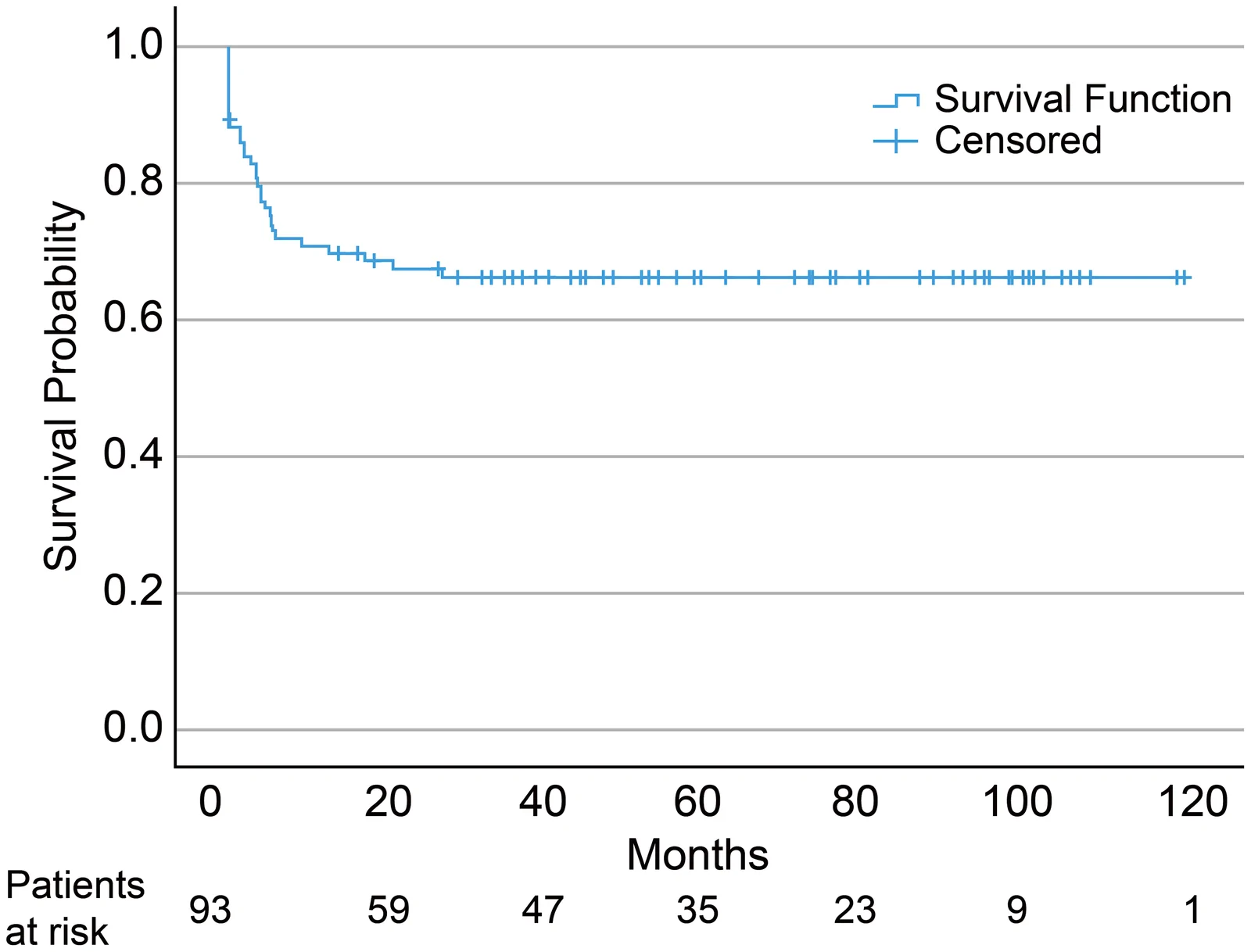
The overall survival rates in this test set.

**Fig. 2:**
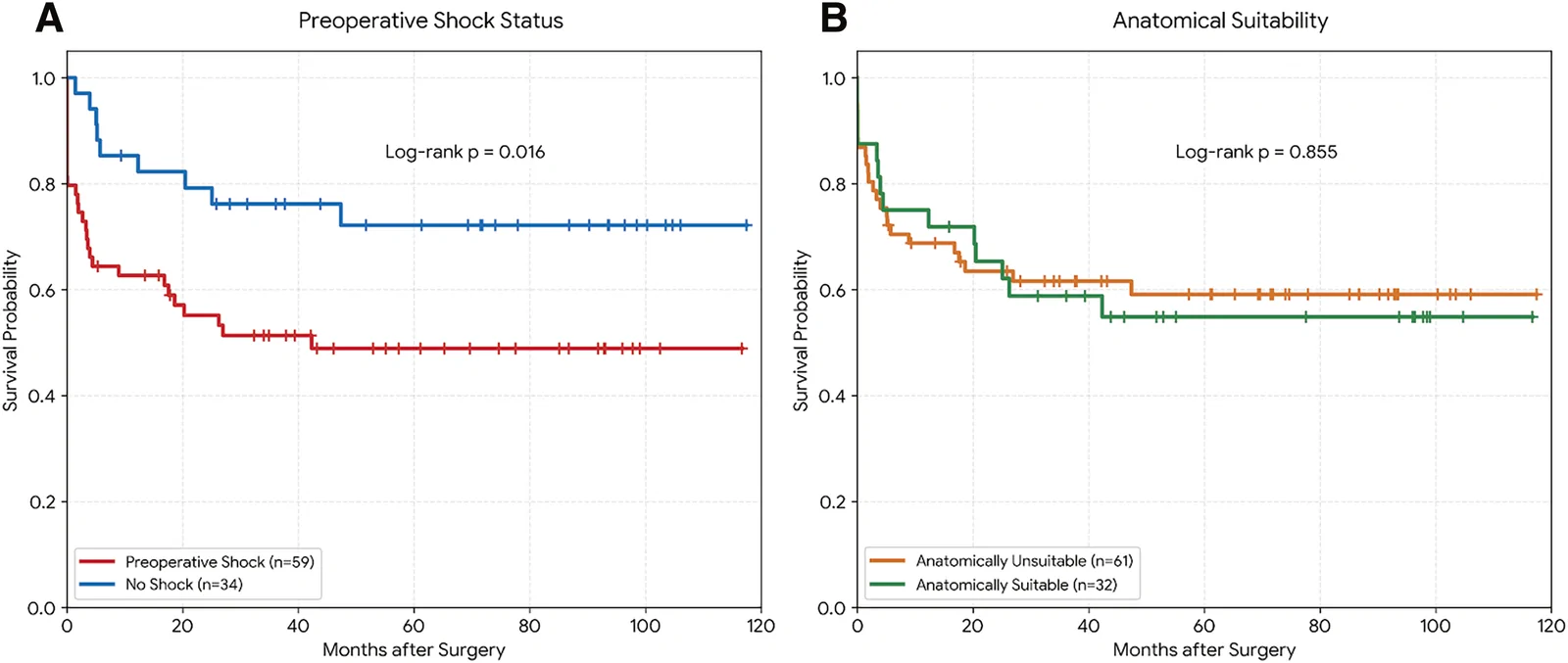
Stratified long-term overall survival curves (Kaplan–Meier estimates) according to baseline subgroups. (**A**) Overall survival stratified by the presence of preoperative shock (severe hypotension defined as systolic blood pressure <80 mmHg). Patients with preoperative shock demonstrated lower early survival, but stable long-term outcomes thereafter. (**B**) Overall survival stratified by anatomical suitability. The survival curves for the anatomically suitable group (n = 32) and the anatomically unsuitable group (n = 61) showed no significant difference, demonstrating that anatomical complexity did not adversely affect long-term survival under the “EVAR-first” protocol (multivariate Cox regression, p = 0.684).

The mean follow-up period was 1386.5 ± 1171.1 (range, 0–3575) days. After the 30-day period, no new pulmonary or renal complications or myocardial infarctions were observed among surviving patients. Endovascular re-interventions were performed in 9 patients (9.7%): 4 with proximal cuffs for type 1a leaks, 3 with external iliac stents, and 2 with coil embolization of the lumbar artery for type 2 leaks. In addition, 1 patient required OSR for an inferior vena cava perforation as a late complication. In the case of inferior vena cava perforation, after EVAR for a ruptured abdominal aortic aneurysm, postoperative CT showed perforation of the inferior vena cava, with blood flow from the inferior mesenteric artery and lumbar artery, resulting in OSR. These details are summarized in **Table 4**. The multivariate Cox proportional hazards regression analysis revealed that preoperative shock was the sole independent predictor of long-term overall mortality (hazard ratio [HR], 2.36; 95% confidence interval [CI], 1.11–5.01; p = 0.025). Other variables, including age (HR, 1.03; 95% CI, 0.99–1.07; p = 0.167), preoperative hemoglobin level (HR, 0.92; 95% CI, 0.81–1.04; p = 0.174), and anatomical unsuitability (HR, 0.87; 95% CI, 0.45–1.69; p = 0.684), were not significantly associated with long-term survival. The overall survival rates at 30 days, 1 year, and 3 years for the entire cohort were 87.1%, 70.9%, and 60.5%, respectively (**Fig. 1**). In the stratified subgroup analysis, the long-term survival curves for the anatomically suitable group (n = 32) and the anatomically unsuitable group (n = 61) were remarkably similar throughout the follow-up period, demonstrating that anatomical complexity did not adversely affect survival under our “EVAR-first” protocol (**Fig. 2**).

**Table 4:** Late outcomes of the “EVAR-first” protocol for ruptured abdominal aortic aneurysms

Variable mean ± SD, or No. (%)	
Mean follow-up, days	1386.5 ± 1171.1
Myocardial infarctions	0
Renal complications	0
Pulmonary complications	0
Endovascular and open re-interventions	9 (9.7)
Period until re-intervention, days	372 ± 279.4
Endovascular re-interventions	9 (9.7)
Proximal cuff for a type 1a endoleak	4 (4.3)
External iliac stents	3 (3.2)
Coil embolization to the lumbar artery for a type II endoleak	2 (2.2)

EVAR, endovascular aortic repair; SD, standard deviation

## Discussion

This study examined the clinical outcomes and feasibility of the “EVAR-first” protocol for treating rAAA in 93 patients over 10 years. The findings showed that the protocol was associated with reduced perioperative mortality, with 30-day, 1-year, and 3-year survival rates of 87.1%, 70.9%, and 60.5%, respectively, demonstrating the feasibility and acceptable clinical outcomes of this protocol in a real-world emergency setting.

The “EVAR-first” protocol for all patients with rAAA may be restricted by some logistical challenges, such as the need for appropriate imaging (often performed at other institutions to assess aortic morphology), the availability of a hybrid operating room capable of performing both OSR and rEVAR, and the availability of a wide variety of grafts. From an international perspective, only 25% of all rAAA underwent EVAR in Sweden^16)^ and in the United States.^17)^ Significant differences in EVAR rates were observed among these institutions. Some institutional constraints have been overlooked; for example, rEVAR in hemodynamically unstable (HD) patients could be performed without preoperative CT using intraoperative imaging.^18)^ Multiple guidelines (the European, AHA/ACC, and Japanese guidelines) clearly state that a CT scan should be performed if the patient’s condition is stable.^13–15)^. In any case, endovascular treatment planning based solely on angiography may not be as appropriate as planning based on CT. Therefore, reconfiguring acute aortic services and establishing standardized institutional protocols may be necessary to implement an EVAR approach for rAAA.^19)^ The rationale for adopting the “EVAR-first” protocol is based on our institutional volume and the extensive proficiency of our multidisciplinary team in endovascular procedures. In our institution, approximately 80% of elective infrarenal AAAs are routinely treated via EVAR. In highly emergent settings involving hemodynamic instability, utilizing this standardized and familiar approach enables the entire surgical and perioperative team to achieve rapid proximal aortic control using EBC with minimal delay. We believe that this standardized workflow minimizes potential human errors under extreme stress, thereby contributing to the favorable survival outcomes observed in our cohort.

Some centers claim that the “OSR-first” strategy yields better outcomes than the “EVAR-first” strategy, even in the era of endovascular repair.^20)^ Furthermore, a previous study reported that the incidence of aortic-related events during the long-term postoperative period is lower with OSR than with the EVAR-first strategy.^21)^ In contrast, another study concluded that the EVAR-first strategy significantly improves early mortality compared to OSR.^22)^ Our results align with those of the IMPROVE trial, which found that the 30-day mortality was similar in the EVAR and OSR groups (35.4% vs. 37.4%, p = 0.62).^2)^ In our study, the 30-day mortality after EVAR was 20.3% in patients with unstable rAAA and 0% in those with stable rAAA. In other studies, EVAR was performed only when patients were hemodynamically stable and anatomically suitable. In contrast, all patients in our study were treated with EVAR, including those with complex anatomical cases. However, most studies and randomized trials do not report mid-term and long-term morbidity and mortality rates, which are important indicators of treatment success. These long-term outcomes may offset the higher cost of endovascular devices and consumables, as shown by van Beek et al.^23)^ and Powell et al.,^2)^ both of which calculated similar average costs within 30 days for the 2 groups. Our findings that 11 patients with rAAA had previously received EVAR due to a late Type Ia endoleak highlight a critical challenge in long-term AAA management. Proximal fixation failure and subsequent neck dilatation over time can result in catastrophic late rupture. This finding underscores the absolute necessity of strict, compliant post-EVAR surveillance. It suggests that an aggressive endovascular strategy, such as immediate proximal cuff extension or conversion maneuvers during the secondary intervention, is mandatory to secure the proximal landing zone and prevent fatal re-rupture in these complex cases.

rAAA remains a significant cause of mortality both before and during hospitalization.^17)^ ACS has been identified as a risk factor for mortality after rEVAR, particularly in cases of EVAR where laparotomy is not routinely performed.^24)^ Many patients have been reported to develop ACS and require decompressive laparotomy. Given the high mortality risk associated with aneurysm rupture, we adopted an aggressive approach to reduce the risk of ACS. We believe that decompressive laparotomy should be performed promptly if ACS is observed or clinically suspected, including immediately after rEVAR for rupture. In our series, ACS was observed in 7 patients, all of whom died within 24 hours of EVAR. Three of these patients required laparotomy. Although ACS may occur more frequently after rEVAR than after OSR, it was not as common in our study, as all patients who underwent rEVAR received muscle relaxant therapy. In our experience, endovascular treatment strategies for rAAA have improved survival by more than 1 year (70.9%) compared to that reported in previous studies.^2)^ The 1-year outcomes of the IMPROVE trial did not show similar results.^25)^ This study indicates that an “EVAR-first” strategy for treating rAAA improves outcomes. Patient selection based on both hemodynamic and anatomical stability influences the choice of treatment, which, in turn, impacts the outcome.^26)^ Although the current database does not assess truncation rates, the fact that patients treated with EVAR were older and had more complications suggests that EVAR was used in patients who would not otherwise have been considered optimal candidates for laparotomy. Thus, primary EVAR centers tend to have better outcomes due to their more frequent use of EVAR. Furthermore, the relationship between volume and outcomes in AAA treatment is well documented.^27)^ For example, in their analysis of the Nationwide Inpatient Sample database, McPhee et al.^28)^ found that the volume of AAA repairs, both intact and ruptured, was predictive of mortality in rAAA treatment. Much of this association was explained by the number of open repairs performed. This finding was similarly observed in a previous Vascunet report on intact repairs, in which the worsening mortality associated with OAR was largely attributed to poorer outcomes in low-volume centers.^29)^ The European Society for Vascular Surgery guidelines recommend that AAA repair should be performed only at hospitals that conduct at least 50 elective infrarenal AAA repairs annually.^30)^ Our data suggest that EVAR for rAAA is beneficial for appropriate candidates. Therefore, the suitability of EVAR for ruptured aneurysms should be carefully determined based on aortic morphology to minimize the risks of procedure-related complications. Compliance with post-EVAR surveillance is an important factor for long-term outcomes in rAAA endovascular strategies. EVAR outcomes have also been shown to depend on institutional volume and experience with this strategy.^27)^

### Study limitations

This study has some limitations. It is a retrospective, non-randomized study based on data from a single center. Further, the sample size was relatively small, which may have introduced selection bias. Therefore, the findings may not be generalizable to other populations. Furthermore, due to the nature of this retrospective review of ultra-emergent cases, precise minute-by-minute records of the time from arrival to aortic balloon occlusion, as well as total blood loss and exact transfusion volumes during the acute phase, were unavailable for a substantial number of patients. Instead, we utilized total operative time as a surrogate indicator of procedural complexity and severity. Furthermore, due to the retrospective nature of this ultra-emergent cohort, precise minute-by-minute records of time to aortic balloon occlusion, total intraoperative blood loss, and exact transfusion volumes during acute resuscitation were unavailable for a substantial number of patients. Although total operative time was evaluated as a surrogate marker for procedural complexity, the lack of granular perioperative hemodynamic and blood loss variables remains an inherent limitation. Therefore, our findings must be interpreted cautiously within the constraints of the available observational data, without extending conclusions beyond the evidence.

## Conclusion

Our 10-year, single-center experience with the “EVAR-first” protocol for rAAA yielded satisfactory perioperative and long-term mortality outcomes, suggesting the potential benefits of this approach. Although the lack of a comparator group is a limitation, previous large-scale registry data, such as the Nationwide Inpatient Sample and Vascunet reports, demonstrate that AAA mortality is closely related to institutional volume and experience. These findings support our results that treating rAAA with highly familiar and proficient techniques in a high-volume center can minimize procedural delays and improve outcomes. However, careful evaluation and close postoperative monitoring are essential because long-term aortic event rates may be higher after EVAR than after OSR. Therefore, reconfiguring acute aortic services and establishing standardized institutional protocols remain crucial for enhancing rAAA management.

## References

[1] Schermerhorn ML, Bensley RP, Giles KA, et al. Changes in abdominal aortic aneurysm rupture and short-term mortality, 1995-2008: a retrospective observational study. Ann Surg 2012; 256: 651–8. 22964737 10.1097/SLA.0b013e31826b4f91 PMC3507435

[2] Powell JT, Sweeting MJ, Thompson MM, et al. Endovascular or open repair strategy for ruptured abdominal aortic aneurysm: 30 day outcomes from IMPROVE randomised trial. BMJ 2014; 348: f7661. 24418950 10.1136/bmj.f7661

[3] Gupta PK, Ramanan B, Engelbert TL, et al. A comparison of open surgery versus endovascular repair of unstable ruptured abdominal aortic aneurysms. J Vasc Surg 2014; 60: 1439–45. 25103257 10.1016/j.jvs.2014.06.122

[4] Marin ML, Veith FJ, Cynamon J, et al. Initial experience with transluminally placed endovascular grafts for the treatment of complex vascular lesions. Ann Surg 1995; 222: 449–65. 7574926 10.1097/00000658-199522240-00004 PMC1234874

[5] Lee CW, Bae M, Chung SW. General considerations of ruptured abdominal aortic aneurysm: ruptured abdominal aortic aneurysm. Korean J Thorac Cardiovasc Surg 2015; 48: 1–6. 25705591 10.5090/kjtcs.2015.48.1.1 PMC4333847

[6] Edwards ST, Schermerhorn ML, O’Malley AJ, et al. Comparative effectiveness of endovascular versus open repair of ruptured abdominal aortic aneurysm in the Medicare population. J Vasc Surg 2014; 59: 575–82.e6. 24342064 10.1016/j.jvs.2013.08.093 PMC4454372

[7] Barnes R, Kassianides X, Barakat H, et al. Ruptured AAA: suitability for endovascular repair is associated with lower mortality following open repair. World J Surg 2014; 38: 1223–6. 24318409 10.1007/s00268-013-2393-y

[8] Mayer D, Aeschbacher S, Pfammatter T, et al. Complete replacement of open repair for ruptured abdominal aortic aneurysms by endovascular aneurysm repair: a two-center 14-year experience. Ann Surg 2012; 256: 688–96. 23095611 10.1097/SLA.0b013e318271cebd

[9] Badger SA, Harkin DW, Blair PH, et al. Endovascular repair or open repair for ruptured abdominal aortic aneurysm: a Cochrane systematic review. BMJ Open 2016; 6: e008391. 10.1136/bmjopen-2015-008391 PMC4762122 26873043

[10] Ali MM, Flahive J, Schanzer A, et al. In patients stratified by preoperative risk, endovascular repair of ruptured abdominal aortic aneurysms has a lower in-hospital mortality and morbidity than open repair. J Vasc Surg 2015; 61: 1399–407. 25752694 10.1016/j.jvs.2015.01.042

[11] Wallace GA, Starnes BW, Hatsukami TS, et al. Favorable discharge disposition and survival after successful endovascular repair of ruptured abdominal aortic aneurysm. J Vasc Surg 2013; 57: 1495–502. 23719035 10.1016/j.jvs.2012.11.089

[12] Ullery BW, Tran K, Chandra V, et al. Association of an endovascular-first protocol for ruptured abdominal aortic aneurysms with survival and discharge disposition. JAMA Surg 2015; 150: 1058–65. 26244272 10.1001/jamasurg.2015.1861

[13] Isselbacher EM, Preventza O, Hamilton Black J 3rd, et al. 2022 ACC/AHA guideline for the diagnosis and management of aortic disease: a report of the American Heart Association/American College of Cardiology joint committee on clinical practice guidelines. Circulation 2022; 146: e334–482. 36322642 10.1161/CIR.0000000000001106 PMC9876736

[14] Wanhainen A, Van Herzeele I, Bastos Goncalves F, et al. Editor’s choice– European Society for Vascular Surgery (ESVS) 2024 clinical practice guidelines on the management of abdominal aorto-iliac artery aneurysms. Eur J Vasc Endovasc Surg 2024; 67: 192–331. 38307694 10.1016/j.ejvs.2023.11.002

[15] Ogino H, Iida O, Akutsu K, et al. JCS/JSCVS/JATS/JSVS 2020 guideline on diagnosis and treatment of aortic aneurysm and aortic dissection. Circ J 2023; 87: 1410–621. 37661428 10.1253/circj.CJ-22-0794

[16] Troëng T, Malmstedt J, Björck M. External validation of the Swedvasc Registry: a first-time individual cross-matching with the unique personal identity number. Eur J Vasc Endovasc Surg 2008; 36: 705–12. 18851920 10.1016/j.ejvs.2008.08.017

[17] Karthikesalingam A, Holt PJ, Vidal-Diez A, et al. Mortality from ruptured abdominal aortic aneurysms: clinical lessons from a comparison of outcomes in England and the USA. Lancet 2014; 383: 963–9. 24629298 10.1016/S0140-6736(14)60109-4

[18] Ohki T, Veith FJ. Endovascular grafts and other image-guided catheter-based adjuncts to improve the treatment of ruptured aortoiliac aneurysms. Ann Surg 2000; 232: 466–79. 10998645 10.1097/00000658-200010000-00002 PMC1421179

[19] Antoniou GA, Ahmed N, Georgiadis GS, et al. Is endovascular repair of ruptured abdominal aortic aneurysms associated with improved in-hospital mortality compared with surgical repair? Interact Cardiovasc Thorac Surg 2015; 20: 135–9. 25281705 10.1093/icvts/ivu329

[20] Seike Y, Yokawa K, Koizumi S, et al. The open-first strategy is acceptable for ruptured abdominal aortic aneurysm even in the endovascular era. Surg Today 2024; 54: 138–44. 37266802 10.1007/s00595-023-02709-6

[21] Yamanaka K, Hamaguchi M, Chomei S, et al. Japanese single-center experience of abdominal aortic aneurysm repair over 20 years: should open or endovascular aneurysm repair be performed first? Surg Today 2023; 53: 1116–25. 36961608 10.1007/s00595-023-02663-3 PMC10519864

[22] Ogino H, Isogai N, Kume N, et al. Evaluating the effectiveness and clinical outcomes of endovascular aneurysm repair-first approach for ruptured abdominal aortic aneurysm in Japan. J Endovasc Ther 2026; 33: 236–43. 38659330 10.1177/15266028241248337

[23] van Beek SC, Conijn AP, Koelemay MJ, et al. Editor’s choice – Endovascular aneurysm repair versus open repair for patients with a ruptured abdominal aortic aneurysm: a systematic review and meta-analysis of short-term survival. Eur J Vasc Endovasc Surg 2014; 47: 593–602. 24746873 10.1016/j.ejvs.2014.03.003

[24] Mehta M, Darling RC 3rd, Roddy SP, et al. Factors associated with abdominal compartment syndrome complicating endovascular repair of ruptured abdominal aortic aneurysms. J Vasc Surg 2005; 42: 1047–51. 16376190 10.1016/j.jvs.2005.08.033

[25] Braithwaite B, Cheshire NJ, Greenhalgh RM, et al. Endovascular strategy or open repair for ruptured abdominal aortic aneurysm: one-year outcomes from the IMPROVE randomized trial. Eur Heart J 2015; 36: 2061–9. 25855369 10.1093/eurheartj/ehv125 PMC4553715

[26] Dubois L, Mayer D, Rancic Z, et al. Debate: Whether endovascular repair offers a survival advantage over open repair for ruptured abdominal aortic aneurysms. J Vasc Surg 2015; 61: 546–55. 25619580 10.1016/j.jvs.2014.11.042

[27] Holt PJ, Poloniecki JD, Gerrard D, et al. Meta-analysis and systematic review of the relationship between volume and outcome in abdominal aortic aneurysm surgery. Br J Surg 2007; 94: 395–403. 17380547 10.1002/bjs.5710

[28] McPhee JT, Eslami MH, Arous EJ, et al. Endovascular treatment of ruptured abdominal aortic aneurysms in the United States (2001-2006): a significant survival benefit over open repair is independently associated with increased institutional volume. J Vasc Surg 2009; 49: 817–26. 19147323 10.1016/j.jvs.2008.11.002

[29] Budtz-Lilly J, Venermo M, Debus S, et al. Editor’s choice – Assessment of international outcomes of intact abdominal aortic aneurysm repair over 9 years. Eur J Vasc Endovasc Surg 2017; 54: 13–20. 28416191 10.1016/j.ejvs.2017.03.003

[30] Moll FL, Powell JT, Fraedrich G, et al. Management of abdominal aortic aneurysms clinical practice guidelines of the European Society for Vascular Surgery. Eur J Vasc Endovasc Surg 2011; 41: S1–58. 21215940 10.1016/j.ejvs.2010.09.011

